# The application of grayscale quantitative analysis in the differential diagnosis of necrosis between cervical lymph node metastases in nasopharyngeal carcinoma and tuberculous lymphadenitis

**DOI:** 10.3389/fonc.2026.1874029

**Published:** 2026-07-28

**Authors:** Yiting Guo, Dan Zhao, Yinan Dong, Yiqiao Lu

**Affiliations:** 1 Department of Ultrasonography, The Second Clinical Medical College, Zhejiang Chinese Medicine University, Hangzhou, China; 2 Department of Ultrasonography, Hangzhou Red Cross Hospital, Tuberculosis Diagnostic and Treatment Center of Zhejiang Province, Hangzhou, Zhejiang, China; 3 Department of Ultrasonography, Hangzhou Xiaoshan Second People’s Hospital, Hangzhou, Zhejiang, China

**Keywords:** cervical tuberculous lymphadenitis, differential diagnosis, nasopharyngeal carcinoma lymph node metastasis, ultrasonic diagnosis, ultrasound grayscale difference

## Abstract

**Objective:**

To investigate the diagnostic value of ultrasound grayscale difference in differentiating necrotic cervical lymph node metastases from nasopharyngeal carcinoma (NPC) and cervical tuberculous lymphadenitis (CTL), and to provide an objective imaging index for clinical differential diagnosis.

**Materials and methods:**

A retrospective study in 2023 enrolled 115 patients with necrotic cervical lymphadenopathy, including 27 with NPC and 88 with CTL. All patients underwent conventional ultrasound, contrast-enhanced ultrasound (CEUS), and ultrasound-guided biopsy. The grayscale difference of necrotic areas was measured by RadiAnt DICOM Viewer. SPSS 25.0 was used for statistical analysis, including univariate and multivariate regression for indicator screening, and receiver operating characteristic (ROC) curves for evaluating diagnostic efficacy.

**Results:**

The ultrasound grayscale difference was 48.07 ± 11.73 in the NPC group, significantly higher than 20.33 ± 9.31 in the CTL group (*p*<0.001). The area under the ROC curve (AUC) of grayscale difference was 0.97, with a cutoff value of 31.5, yielding a sensitivity of 96.3% and specificity of 90.9%. The multivariate regression model achieved an AUC of 0.97, with sensitivity of 95.5% and specificity of 92.6%. Age and gender differed significantly between groups (*p*<0.05), while lymph node diameters showed no significant difference (*p*>0.05).

**Conclusion:**

Ultrasound grayscale difference has high diagnostic value in distinguishing necrotic cervical lymph node metastases of NPC from CTL, and can serve as a reliable quantitative imaging indicator. The multivariate regression model further improves diagnostic accuracy and supports clinical decision-making.

## Introduction

Lymph nodes serve as a crucial immune defense barrier in the human body, with pathological changes involving various mechanisms. Among these, tumor metastasis and infectious destruction pose significant challenges in clinical differential diagnosis. Nasopharyngeal carcinoma, a prevalent malignancy in the southern regions of China, exhibits a notably high incidence of cervical lymph node metastasis, reaching 60%−80% (1), and often represents a primary clinical manifestation at the time of patient presentation. The comprehensive management of nasopharyngeal carcinoma (NPC), particularly in patients with cervical lymph node involvement, requires a multidisciplinary approach encompassing accurate diagnosis, appropriate staging, and optimal treatment strategies (2). In contrast, the incidence of cervical lymphadenitis due to tuberculosis is on the rise globally amid a resurgence of tuberculosis cases (3). Some patients present solely with painless lymphadenopathy, which overlaps clinically with the manifestations of nasopharyngeal carcinoma metastases. Furthermore, both conditions are prone to lymph node necrosis, leading to misdiagnosis in approximately 15%−27% of cases upon initial assessment (4, 5).

Although numerous studies have indicated that contrast-enhanced ultrasound technology can effectively assess the extent of necrosis within lymph nodes (6, 7), it does not facilitate the analysis of characteristics within the necrotic regions, and some patients are unable to undergo the invasive procedure of contrast agent injection. In the present study, contrast-enhanced ultrasound (CEUS) was employed primarily to confirm the presence of necrosis and to guide biopsy targeting, rather than as a standalone diagnostic modality for differentiating between the two conditions. In contrast, grayscale ultrasound, as a fundamental imaging technique, can reflect tissue characteristics through the intensity of echoes. By utilizing the grayscale difference (the relative difference in grayscale values between the lesion area and normal tissue), this technique can quantify those characteristics (8, 9). Furthermore, with the increasing availability of high-resolution ultrasound and advancements in computer technology, the application of ultrasound grayscale differences has been extensively researched and promoted across various disease diagnoses (8, 10). This trend toward quantitative imaging analysis aligns with the broader movement in diagnostic imaging toward objective and precise cancer diagnosis, as exemplified by the integration of artificial intelligence with digital imaging technologies in other fields (11). However, reports on its application in necrotic lymph node diseases remain rare.

This study conducts a comparative analysis of the ultrasound grayscale difference between necrotic regions in lymph node metastasis of nasopharyngeal carcinoma and those in cervical lymphadenitis tuberculosis. The findings elucidate the distinguishing characteristics of necrotic areas in both conditions, thereby providing a basis for the differential diagnosis using grayscale ultrasound. Furthermore, it offers objective indicators for clinical differentiation.

## Materials and methods

### Ethical approval

Ethical approval was obtained from the institutional review board, and written informed consent was obtained from all patients.

### Patients

This retrospective study included patients diagnosed with either lymph node metastasis from nasopharyngeal carcinoma (NPC) group or tuberculous lymphadenitis (CTL) group at our institution between January and December 2023. The inclusion criteria were as follows: (1) presence of cervical lymphadenopathy; (2) a definitive diagnosis of lymph node metastasis from nasopharyngeal carcinoma or cervical lymphadenitis tuberculosis, confirmed by pathological or laboratory examinations; (3) no history of allergic conditions and the ability to tolerate CEUS; (4) no underlying diseases that would interfere with the study and the ability to undergo ultrasound-guided fine needle aspiration biopsy. Exclusion criteria included: (1) incomplete data; (2) unclear diagnostic conclusions; (3) presence of other malignancies, immunodeficiency disorders, or prior radiotherapy/chemotherapy before ultrasound examination; (4) poor quality of ultrasound images that prevented accurate grayscale ratio measurements.

**Figure 1:**
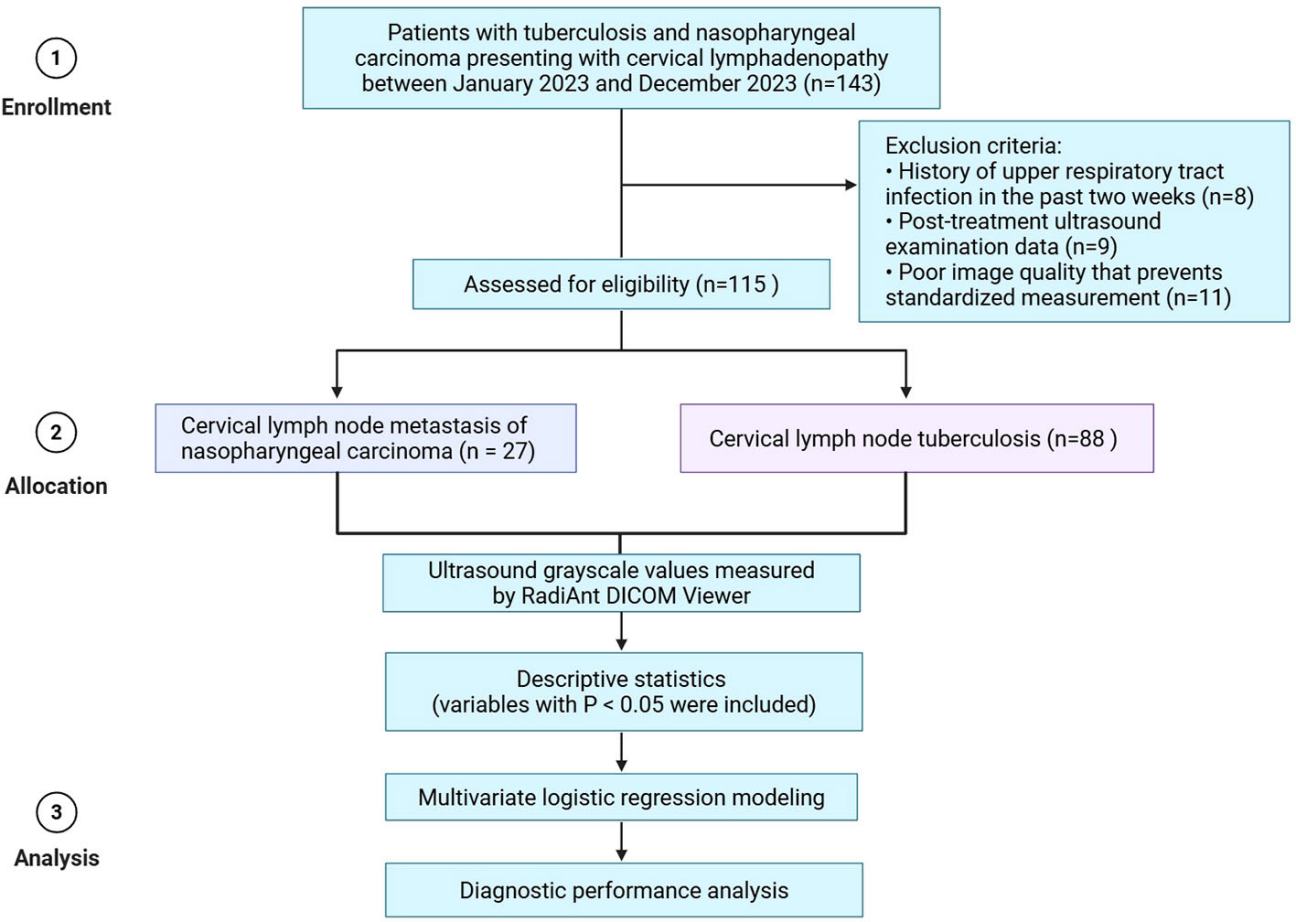
Flowchart of patient enrollment and grouping.

A total of 115 cases were included in the study, with 27 patients in the NPC group and 88 patients in the CTL group (**Figure 1**). The NPC group consisted of 18 males and 9 females, aged between 20 and 85 years, with a mean age of 51.07 ± 14.89 years. The CTL group comprised 58 males and 30 females, aged between 14 and 76 years, with a mean age of 42.06 ± 16.02 years.

### Ultrasound examination and biopsy by puncture

**Figure 2:**
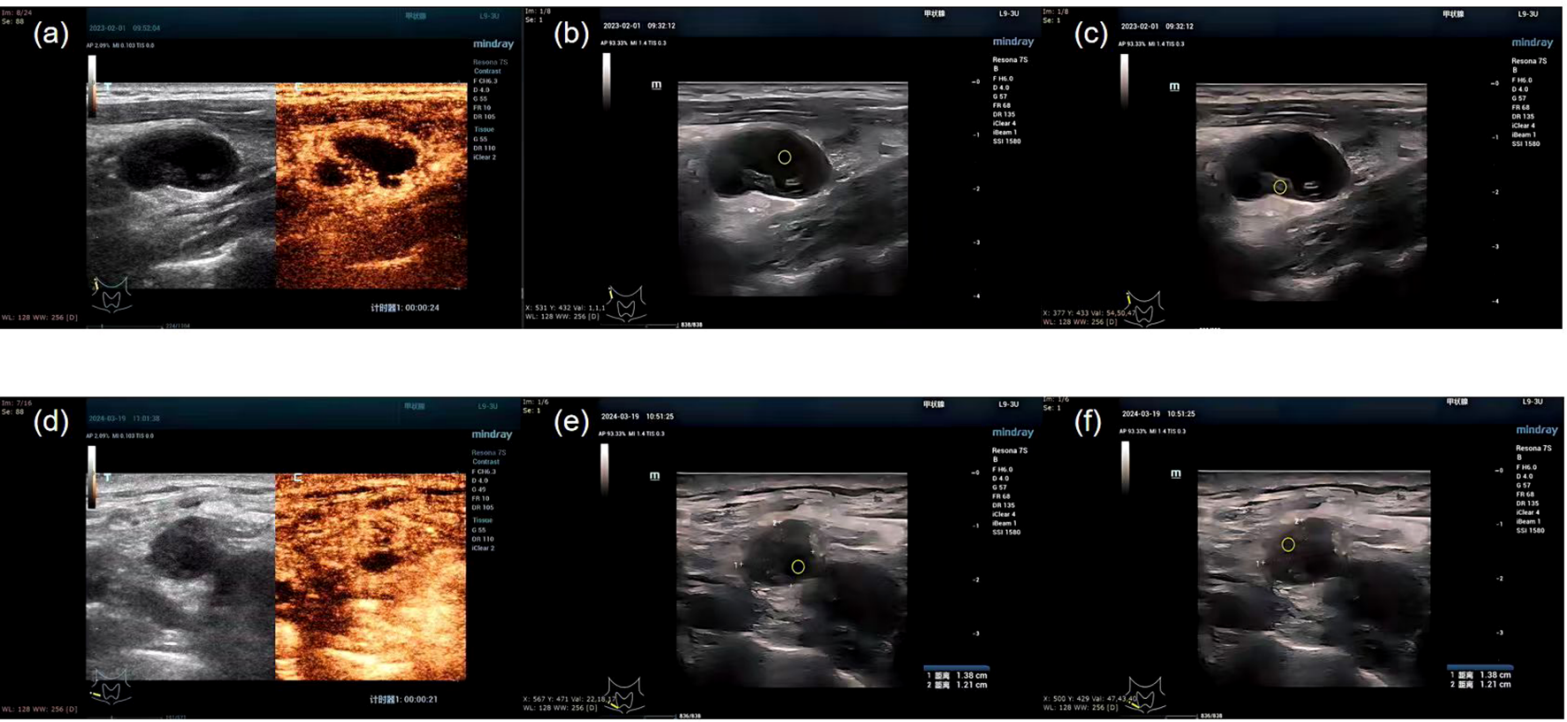
Representative grayscale and contrast-enhanced ultrasound images of necrotic cervical lymph nodes. **(a–c)** A patient with pathologically confirmed nasopharyngeal carcinoma cervical lymph node metastasis: **(a)** Contrast-enhanced ultrasound showing heterogeneous enhancement of the lymph node with a large necrotic area; **(b)** Grayscale ultrasound with region of interest (ROI) placed in the necrotic area for grayscale quantitative analysis; **(c)** ROI placed in the non-necrotic solid area of the same lymph node. **(d–f)** A patient with pathologically confirmed cervical tuberculous lymphadenitis: **(d)** Contrast-enhanced ultrasound showing ring-like enhancement of the lymph node with central necrosis; **(e)** Grayscale ultrasound with ROI placed in the necrotic area; **(f)** ROI placed in the non-necrotic solid area of the lymph node.

Diagnosis was conducted using the Mindray Resona 7S color Doppler ultrasound system (Shenzhen Mindray Bio-Medical Electronics Co., Ltd., Shenzhen, China), employing a typical ultrasound examination with an L9–3 broadband linear array transducer (frequency 3–9 MHz). Patients were positioned supinely with the neck comprehensively exposed, and a systematic assessment was performed to identify the maximum lymph node, recording the corresponding ultrasound findings. Subsequently, a contrast-enhanced ultrasound examination was performed using an L9–3 broadband linear array transducer (frequency 3–9 MHz) employing a low mechanical index (0.06) pulsed harmonic imaging technique. The contrast agent, SonoVue (Bracco, Milan, Italy), was initially diluted with 5 ml of saline, agitated, and then administered via the antecubital vein as a bolus of 2.4 ml, followed by a 10ml saline flush. The time of contrast agent injection was duly recorded, and real-time dynamic monitoring of lymph node perfusion was conducted using dual-phase contrast imaging, with continuous observation for 3 minutes while storing the entire imaging process. The CEUS findings were primarily used for two purposes: (1) to confirm the presence and extent of necrosis within the lymph nodes, ensuring that only cases with definite necrotic areas were included for grayscale analysis; and (2) to guide the selection of optimal puncture targets for ultrasound-guided fine-needle aspiration biopsy, with the viable peripheral regions (rather than the necrotic center) being preferentially targeted to maximize diagnostic yield. The CEUS perfusion patterns were not used as independent diagnostic criteria in this study, nor were they incorporated into the final statistical model, as the primary objective was to evaluate the diagnostic value of grayscale difference derived from conventional ultrasound. The maximum diameter (long axis) was analyzed, computed, and documented. The ultrasound grayscale difference was measured using the RadiAnt DICOM Viewer (2023.1), selecting the necrotic regions within the lesions as the region of interest (ROI) and using the surrounding viable tissue as the reference (**Figure 2**). The difference in gray values between the ROI and the reference area was calculated, with the average of three measurements recorded. The entire process was analyzed, evaluated, and documented by two experienced radiologists; any discrepancies were resolved through discussion involving a third senior radiologist. Following the contrast-enhanced ultrasound examination, fine-needle aspiration biopsy was promptly performed under ultrasound guidance using the L9–3 transducer. The biopsy specimens underwent pathological and laboratory investigations, leading to a final diagnosis of lymph node metastasis from nasopharyngeal carcinoma or cervical lymphadenitis tuberculosis, corroborated by acid-fast staining, next-generation sequencing, or Xpert MTB/RIF assay in laboratory investigations. The selection of the puncture pathway was determined by two senior radiologists based on analyses from both conventional ultrasound and contrast-enhanced ultrasound; in the event of disagreement, a third senior radiologist was consulted.

### Statistical analysis

Data processing was performed using the SPSS statistical software package (version 25.0; SPSS Inc., Chicago, IL). For categorical data, the Chi-square test and Fisher’s exact test were employed to assess differences between the NPC group and the control (CTL) group. Continuous data were analyzed using the t-test. A p-value<0.05 was considered statistically significant. Two-dimensional ultrasound characteristics of the lesions, including the long and short axes, ultrasound grayscale differences, and the demographic characteristics of the patients (such as age and gender), were treated as independent variables in a univariate analysis. Statistically significant ultrasound characteristic variables were subsequently included in a binary logistic multivariable regression analysis. Receiver operating characteristic (ROC) curves were generated with a significance level set at α=0.05.

## Results

**Figure 3:**
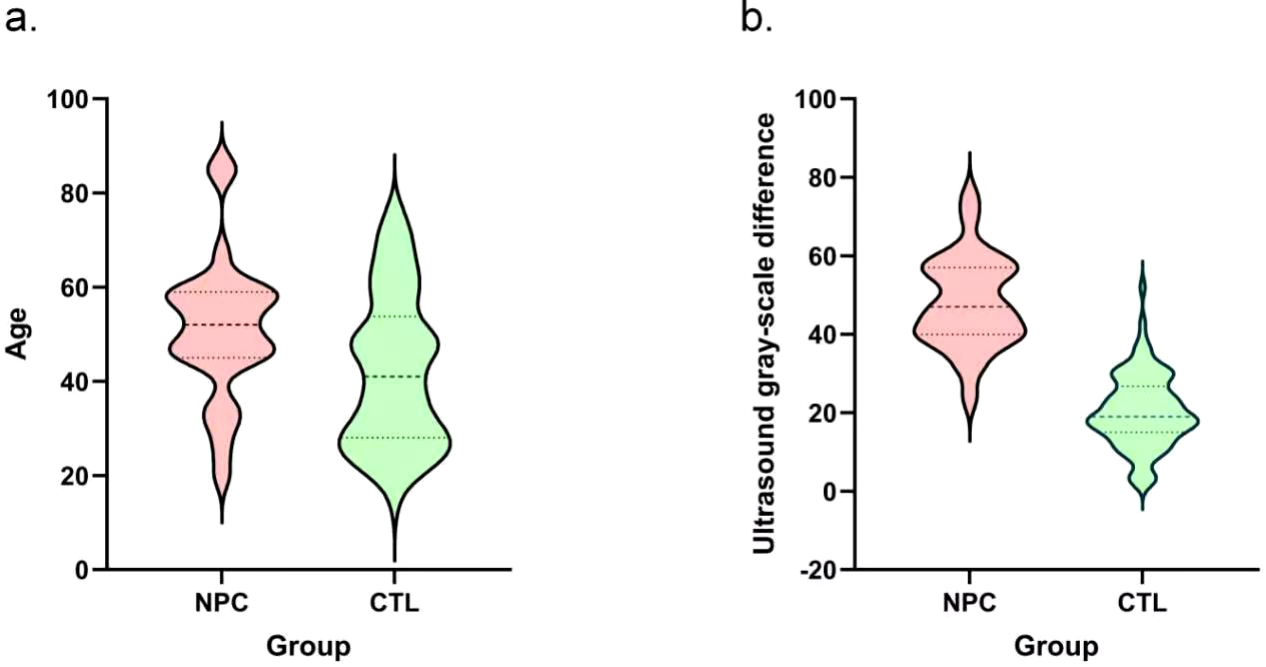
**(a)** Comparison of age distribution between nasopharyngeal carcinoma (NPC) group and cervical tuberculous lymphadenitis (CTL) group, p < 0.05. **(b)** Comparison of ultrasound grayscale difference between nasopharyngeal carcinoma (NPC) group and cervical tuberculous lymphadenitis (CTL) group, p < 0.001.

This retrospective study included a total of 115 patients with cervical lymphadenopathy, categorized based on pathological diagnoses into the nasopharyngeal carcinoma lymph node metastasis group (referred to as the NPC group, n=27) and the cervical lymphadenitis due to tuberculosis group (referred to as the tuberculosis group, n=88). The baseline characteristics of the two groups, ultrasound parameters, and the results of the diagnostic efficacy analysis are presented as follows (**Table 1**). The analysis revealed several significant findings. Firstly, the average age in the NPC group was higher than that in the CTL group, with the NPC group displaying an average age of 51.07 ± 14.89 years compared to 42.06 ± 16.02 years in the CTL group, a difference that was statistically significant (*t*=2.599, *p*=0.011, **Figure 3A**). Furthermore, the male-to-female ratio in the NPC group was 1.6:1, whereas in the tuberculosis group it was 1:2, with this difference also being statistically significant (*χ²*=7.057, *p*=0.003). No significant differences were observed between the two groups regarding the long and short diameters of the lymph nodes (*p*>0.05). Additionally, the ultrasound grayscale difference in the NPC group was significantly higher than that in the CTL group (48.07 ± 11.73 vs 20.33 ± 9.31), with a statistically significant difference (*t*=12.713, *p*<0.001, **Figure 3B**).

**Table 1:** Baseline characteristics of the patients and ultrasound features.

Variable	NPC	CTL	t/x²	P-value
**Gender**			9.016	0.003
Female	9 (33.3%)	58 (65.9%)		
Male	18 (66.7%)	30 (34.1%)		
**Mean age (years)**	51.07 ± 14.89	42.06 ± 16.02	2.599	0.011
**Long Axis (cm)**	2.75 ± 0.78	3.22 ± 1.48	-1.719	0.088
**Short Axis (cm)**	1.59 ± 0.38	1.83 ± 0.95	-1.292	0.199
**Ultrasound Grayscale Difference**	48.07 ± 11.73	20.33 ± 9.31	12.713	< 0.001

NPC, nasopharyngeal carcinoma with cervical lymph node metastasis; CTL, cervical tuberculous lymphadenitis.

On contrast-enhanced ultrasound, the NPC group predominantly exhibited heterogeneous enhancement with irregular peripheral rim enhancement and central non-enhancing necrotic areas (23/27, 85.2%), while the CTL group more frequently showed peripheral rim enhancement with central non-enhancing caseous necrosis (72/88, 81.8%). These CEUS findings were consistent with previously reported patterns (19–22) and served to confirm the presence of necrosis in all included cases, thereby validating the selection of necrotic regions for grayscale quantitative analysis. However, as the CEUS perfusion characteristics were not the focus of this study, they were not subjected to quantitative statistical analysis.

In the binary logistic multivariable regression analysis, variables that demonstrated statistical differences in the univariate analysis were included. The results of the multivariable regression analysis indicated that multiple characteristics could serve as discriminative markers between the two groups. A logistic regression model was constructed using all the included variables to predict the membership of the groups. The model is expressed as follows: Logistic (Y)=11.050+0.190x_1_−0.038x_2_−0.243x_3_.

**Figure 4:**
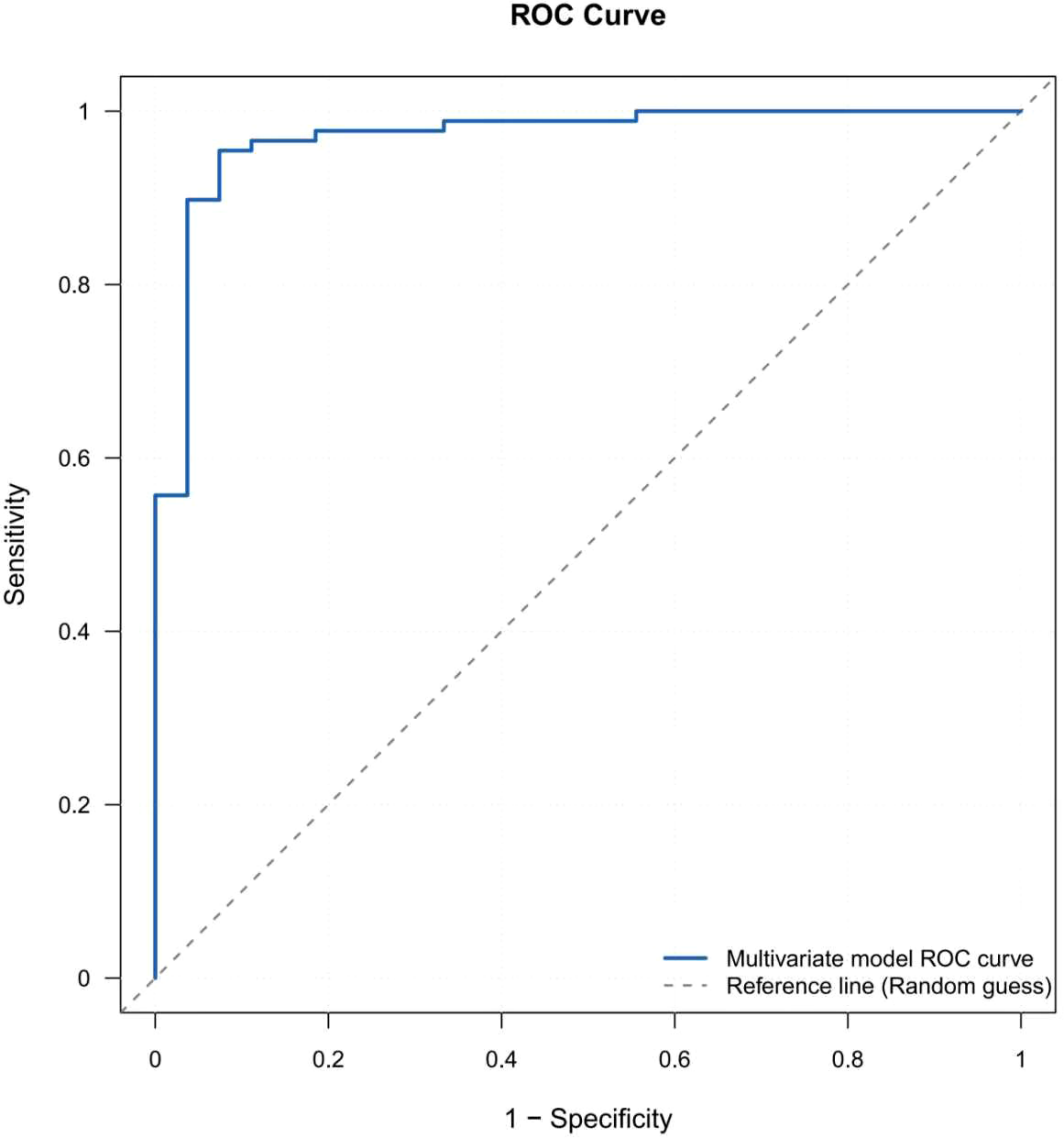
Receiver operating characteristic (ROC) curve of the multivariate logistic regression model for differentiating nasopharyngeal carcinoma lymph node metastasis from cervical tuberculous lymphadenitis. The area under the curve (AUC) was 0.970 (95% confidence interval: 0.934–1.000; p < 0.001), indicating excellent diagnostic performance.

The logistic regression equation was utilized to evaluate the discriminative capability between the two conditions. To consolidate these features, a ROC curve was plotted (**Figure 4**), with the AUC measured at 0.970, indicating a high overall predictive accuracy of the model. The standard error was 0.018, and the result was statistically significant (*p*<0.001). The 95% confidence interval for the AUC ranged from 0.934 to 1.000. Using the Youden index, the maximum value of 0.5914 correlated with a regression equation value of Y=3.873. By incorporating the indicators into the regression equation, Y values could be calculated. When Y≥3.873, the prediction is that the lymph node is a metastasis of nasopharyngeal carcinoma; conversely, when Y ≤ 3.873, it is predicted to be a case of lymphadenitis due to tuberculosis. This predictive method demonstrated a sensitivity of 95.5%, specificity of 92.6%, accuracy of 94.8%, positive predictive value of 97.7%, and negative predictive value of 86.2%.

## Discussion

NPC is a prevalent malignant neoplasm in southern China, often diagnosed with cervical lymph node metastasis in 60%-80% of cases. The primary mechanism for this metastasis is the rich lymphatic network in the nasopharynx, enabling cancer cells to invade cervical lymph nodes via lymphatic channels or bloodstream, leading to metastatic lesions (12–14). Similarly, tuberculous lymphadenitis (TL), the most common form of extrapulmonary tuberculosis, also frequently involves the cervical region. Mycobacterium tuberculosis disseminates to the cervical lymph nodes, where it induces granuloma formation and caseous necrosis, disrupting normal lymph node architecture (15–17). Necrotic areas are often present within the lesions of both pathologies, appearing as anechoic or hypoechoic regions on ultrasound imaging (18–20), which leads to a misdiagnosis rate of approximately 15%-27% at the initial presentation (5). Therefore, distinguishing between NPC with cervical lymph node metastasis and cervical lymphadenitis due to tuberculosis is crucial for proper diagnosis and treatment.

CEUS provides real-time monitoring of tissue perfusion and hemodynamic characteristics, such as rapid influx and efflux or gradual filling and drainage. Although CEUS is effective in assessing necrosis within lymph nodes, it primarily provides information about tissue perfusion and lacks the ability to evaluate the necrotic regions themselves (21–24). Additionally, some patients are unable to undergo contrast agent injection. On the other hand, ultrasound elastography measures tissue elasticity, with malignant lesions generally being stiffer than benign lesions due to active cellular proliferation and significant stromal fibrosis. However, necrotic areas within lymph nodes often present as low stiffness, making it difficult to distinguish necrosis from other tissue changes based solely on elasticity modulus or color differences in elastography images (25–27). Both techniques have demonstrated clinical value but also possess inherent limitations. It should be noted that the present study did not directly compare the diagnostic performance of grayscale difference with that of CEUS or elastography; therefore, no direct conclusion regarding the superiority of any single modality can be drawn from our data.

Digital imaging advancements have led to the use of grayscale ultrasound to assess tissue characteristics. Grayscale ultrasound reflects tissue properties based on echo intensity, and the grayscale difference, which quantifies the difference in grayscale values between pathological and normal tissues, can be particularly useful in differentiating necrotic regions within lymph nodes (8, 9). Consequently, we introduce the ultrasound grayscale difference technique to conduct a comparative analysis of the necrotic regions in metastatic cervical lymph nodes associated with nasopharyngeal carcinoma and those affected by cervical lymphadenitis due to tuberculosis, thereby revealing the differential characteristics of the necrotic areas. By establishing an appropriate threshold for the grayscale difference, we can significantly enhance the diagnostic efficacy between the two conditions, further improving the accuracy of differential diagnosis and providing a reliable basis for the selection of clinical treatment strategies.

Although gender and age differences exist, their diagnostic value is limited (AUC=0.65), and they cannot serve as independent diagnostic criteria. Additionally, there is no significant difference in lymph node size between NPC and CTL (*p*=0.118), as both conditions can cause lymph node enlargement. Nasopharyngeal carcinoma-lymph node metastasis (NPC-LNM) may present as small early metastatic foci due to slower tumor cell proliferation, whereas CTL can cause node enlargement due to inflammatory exudation and granuloma formation (28, 29). In contrast, CTL may lead to lymph node enlargement due to inflammatory exudation and granuloma formation induced by Mycobacterium tuberculosis. Furthermore, in the later stages of the disease, CTL may result in the liquefaction of caseous necrotic tissue, causing a reduction in lymph node size (30), which further complicates size-based differentiation. Consequently, the traditional ultrasound diagnostic approach reliant on “lymph node size” exhibits evident limitations, necessitating the incorporation of quantitative indicators, such as the grayscale difference, to enhance diagnostic accuracy.

This study found that the grayscale difference in the NPC group was significantly higher than that in the CTL group (*p*<0.001), with a ROC curve AUC reaching 0.97. The optimal cutoff value of 31.5 yielded a sensitivity of 96.3% and a specificity of 90.9%, indicating that the ultrasound grayscale difference possesses exceptional discriminatory value. This outcome may be closely related to the underlying pathological differences between the two conditions. Within metastatic cervical lymph nodes of NPC, there is a marked discrepancy in cell density between necrotic and non-necrotic regions (13). In non-necrotic areas, tumor cells are densely arranged with a high nuclear-to-cytoplasmic ratio (large nuclei and scant cytoplasm), accompanied by minimal intercellular stroma and low degrees of fibrosis (31). In contrast, the necrotic regions exhibit an extremely low cell density, as these areas represent the necrotized segments of the tumor, where cellular architecture disintegrates, resulting in cell density approaching zero (32). Additionally, research has demonstrated that regions of high cell density typically generate stronger ultrasonic scattering (33), leading to higher ultrasound grayscale values. Consequently, the ultrasound grayscale difference for NPC-LNM (the grayscale value of non-necrotic regions minus that of necrotic regions) is significantly elevated (with an average of 48.07 in this study). Furthermore, the distribution of grayscale differences in NPC-LNM is relatively concentrated around 47.0 (40.0, 56.5), likely related to the relatively uniform pathological typing of NPC tumor cells, predominantly consisting of non-keratinizing carcinoma, with minimal variation in cell proliferation activity and stromal composition, resulting in stable echo intensities (34). However, direct pathological correlation with ultrasound grayscale values was not performed in the present study, and the above interpretation remains speculative and warrants further histopathological validation.

The pathological characteristics of cervical tuberculous lymphadenitis (CTL) include the formation of caseating necrotizing granulomas, induced by the aggregation and fusion of macrophages due to Mycobacterium tuberculosis, resulting in the formation of Langhans giant cells. This is subsequently followed by macrophage apoptosis and necrosis, culminating in the formation of acellular caseous necrotic tissue (35, 36). The primary components of caseous necrotic tissue consist of necrotic cellular debris, lipids, and inflammatory exudates, which are characterized by low acoustic impedance and a weak ultrasound reflection, leading to diminished grayscale values (36, 37). However, the material characteristics of this necrotic tissue result in grayscale values that surpass those of areas undergoing complete liquefactive necrosis, such as tumor metastases. Moreover, despite the presence of a limited cellular component within the granulomatous regions of CTL, the loose arrangement of cells contributes to an echo intensity that remains lower than that of normal muscular layers (30, 38). These two factors collectively result in a significantly lower grayscale difference of the necrotic regions in CTL compared to nasopharyngeal carcinoma (NPC) cases, with an average value of 20.33 observed in this study. Additionally, we noted a broader distribution range of grayscale difference in the necrotic areas of CTL, spanning from 19.0 (15.0, 26.3), which may be attributed to varying degrees of caseous necrosis among the enrolled cases. In the early stages of necrosis, the presence of inflammatory exudates within the necrotic tissue may dilute the necrotic material, resulting in lower grayscale values (39). Conversely, in later stages, as fluids are absorbed by the tissue, the necrotic material becomes denser and even solidified, leading to a gradual increase in grayscale values (30). Furthermore, the necrotic regions coexist in an interspersed manner with the granulomatous tissue, and certain necrotic areas may exhibit incomplete necrosis, with traces of granulomatous tissue remaining (40, 41). These factors may contribute to an elevation of grayscale values in the necrotic regions due to insufficient ultrasound resolution and partial volume effects, consequently resulting in a relative decrease in grayscale difference. Despite some degree of variability, the grayscale difference of CTL are consistently lower than those in the NPC group, thereby facilitating effective differentiation. Nevertheless, direct histopathological verification of the correspondence between grayscale values and specific tissue components was not conducted, and this mechanistic interpretation requires confirmation in future studies. Furthermore, by using region of interest (ROI) quantification, the method reduces subjective variability and ensures strong inter-observer agreement (Kappa=0.82).

However, this study has limitations. As a single-center retrospective study, the sample size was relatively small, particularly for the NPC group (27 cases), which could introduce selection bias. And the sample size imbalance may limit the generalizability of the findings and precluded the use of propensity score matching due to the limited number of NPC cases. Additionally, we compared grayscale differences with normal perfused tissue adjacent to necrotic regions, which may be influenced by underlying pathological changes in the lymph nodes. The study focused only on NPC-LNM and CTL, excluding other necrotic pathological conditions like necrotizing lymphadenitis. Future studies with larger sample sizes and broader pathological conditions are necessary to validate these findings and further refine diagnostic accuracy.

## Conclusion

In conclusion, this study affirmatively demonstrates the significant value of ultrasonic grayscale difference in distinguishing between nasopharyngeal carcinoma lymph node metastases and cervical lymphadenitis due to tuberculosis. Additionally, the research employed statistical methodologies to ascertain the optimal threshold for differentiating the two conditions, thus providing objective criteria for differential diagnosis.

## Data Availability

The raw data supporting the conclusions of this article will be made available by the authors, without undue reservation.
